# Children and Careers: How Family Size Affects Parents’ Labor Market Outcomes in the Long Run

**DOI:** 10.1007/s13524-017-0612-0

**Published:** 2017-09-06

**Authors:** Sara Cools, Simen Markussen, Marte Strøm

**Affiliations:** 1 Institute for Social Research, Postboks 3233 Elisenberg, 0208 Oslo, Norway; 2 Ragnar Frisch Centre for Economic Research, Gaustadalléen 21, 0349 Oslo, Norway

**Keywords:** Family size, Labor supply, Career, IV estimation, Parenthood

## Abstract

We estimate the effect of family size on various measures of labor market outcomes over the whole career until retirement, using instrumental variables estimation in data from Norwegian administrative registers. Parents’ number of children is instrumented with the sex mix of their first two children. We find that having additional children causes sizable reductions in labor supply for women, which fade as children mature and even turn positive for women without a college degree. Among women with a college degree, there is evidence of persistent and even increasing career penalties of family size. Having additional children reduces these women’s probability of being employed by higher-paying firms, their earnings rank within the employing firm, and their probability of being the top earner at the workplace. Some of the career effects persist long after labor supply is restored. We find no effect of family size on any of men’s labor market outcomes in either the short or long run.

**Electronic supplementary material:**

The online version of this article (10.1007/s13524-017-0612-0) contains supplementary material, which is available to authorized users.

## Introduction

Parenthood is a major cause of reduced labor supply for women. It is therefore a likely candidate for explaining the persisting career gap between women and men. Although the gender gap in wages has been steadily decreasing over the last decades—largely driven by the catching up, and even surpassing, of women’s educational level relative to men’s—women’s career paths are less steep than men’s (Blau and Kahn 2016; Olivetti and Petrongolo 2016).

Women are particularly underrepresented at the top (Bertrand and Hallock 2001; Bertrand et al. 2010). A substantial share of the persistent difference in men’s and women’s careers can be traced to differences in career breaks and to shorter work hours for women compared with men (Blau and Kahn 2016). The consequences of reduced labor supply are larger in higher-paying occupations (Adda et al. 2017; Goldin 2014).

A growing body of literature has documented substantial effects of parenthood and family size on female labor supply (Angrist and Evans 1998; Lundberg and Rose 2000; Lundborg et al. 2014). If the lagging behind of female wages is a consequence of family-related career breaks and shorter work hours, there should also be an effect of children on career measures. Yet, the literature on career effects has relied mainly on correlations in the cross section or on panel data estimation (Budig and England 2001; Harkness and Waldfogel 2003; Korenman and Neumark 1992; Wilde et al. 2010). Evidence of career effects of children that credibly addresses selection and endogeneity issues remains scarce.

In this study, we aim to estimate the effect of family size on a comprehensive set of labor supply and career measures in both the short and the long run. We address the problem of endogeneity in the family size decision by using the sex composition of the first two children as an instrumental variable (IV) for the total number of children (Angrist and Evans 1998). Because children’s sex is randomly determined and some parents seemingly hold a preference for having children of both sexes—a fact long noted by demographers (Ben-Porath and Welch 1976; Gini 1951)—having two same-sex children increases the probability of further childbearing in a way that is unrelated to unobservable factors that influence parents’ labor market outcomes. Children’s sex composition can also be observed over a long period, enabling us to cover the whole working career of Norwegian men and women.

We study the effect of an increase in family size on labor supply (income, labor force participation, and weekly work hours) and on career outcomes, such as earnings rank (of both the individual and her firm) and the probability of being the top earner at the workplace. The study sample contains all Norwegian families with a second child born in 1970–2001, and we consider outcomes up until 40 years after the birth of the second child. We run separate estimations for each year since the birth of the second child in order to map out the full trajectory of effects.

The career breaks and shorter work hours caused by taking care of children would expectedly be larger in human capital–intensive occupations (Mincer and Polachek 1974) or where the earnings potential is larger and wage paths are steeper (Adda et al. 2017). We split the sample into college graduates and non–college graduates to allow for different responses in these two groups.

Our findings show significant negative effects of additional children on labor supply in the short to medium run. In the long run, however, labor supply is restored. The effects on career outcomes are more persistent. We find clear evidence that having additional children impedes women’s careers in the longer run and that these effects are concentrated among college-educated women. Women with a college degree have a smaller probability of being employed in a high-paying firm throughout the rest of their career. There is, however, no long-term effect on the probability of being the top earner or on the individual’s earnings rank within the firm. The results therefore suggest that family size affects women’s type of employment, given that women with more children work in less-prestigious places of work but seem to have the same within-firm status as women with fewer children. We find no effects of family size on men’s career prospects.

## Previous Literature

The literature on the effect of parenthood on labor market outcomes generally takes Becker’s (1981) theories of household specialization and human capital development as a starting point. Childbearing and child rearing have a direct effect on (mothers’) labor supply, both through periods out of the labor market and through periods of reduced working hours, thereby impeding mothers’ accumulation of human capital. In addition, Becker’s (1985) theory of a conflict between effort in home production and effort at work implies that among women working the same number of hours, women with more children will be less productive and consequently less successful in the labor market.

In line with Becker’s reasoning, the surpassing of women’s educational level relative to men’s during the last decades has been followed by a steady decrease in the gender gap in wages. At the same time, it has become increasingly clear that women’s career paths are less steep than men’s, particularly at the high end of the wage distribution (Bertrand and Hallock 2001; Bertrand et al. 2010; Blau and Kahn 2016; Olivetti and Petrongolo 2016). Cross-sectional and panel data studies have suggested that a substantial share of the persistent difference in men’s and women’s careers can be explained by differences in career breaks and by women’s shorter work hours relative to men’s (Anderson et al. 2002; Blau and Kahn 2016; Budig and England 2001; Waldfogel 1997). The cost of reduced labor supply is larger in higher-paying occupations, in which pay depends nonlinearly on presence at work (Goldin 2014) and the average wage profile is steeper (Adda et al. 2017).

The present study contributes to the existing literature on how parenthood affects labor market outcomes in several ways. First, we bring long-term evidence to the growing literature on labor supply effects of children that uses IV techniques. The most-used instrumental variables are sex composition and twinning (Angrist and Evans 1998; Bronars and Grogger 1994; Cruces and Galiani 2007; Jacobsen et al. 1999) for higher-order births, and miscarriage, *in vitro* fertilization (IVF) treatment, and infertility (Agüero and Marks 2011; Cristia 2008; Hotz et al. 1997; Lundborg et al. 2014; Markussen and Strøm 2015; Miller 2011) for both entry into parenthood and births at higher margins, and to estimate the consequences of delaying childbirth. Most studies have found a negative effect on women’s labor force participation in the short to medium run in developed countries. Insofar as men’s labor market outcomes are being studied, there are comparably small and insignificant effects of family size on labor supply (Angrist and Evans 1998; Cools 2013; Hirvonen 2009). Attempts at estimating the duration of the effects on labor supply in the IV setting suggest that the reduction in women’s labor force participation is temporary (Angelov and Karimi 2012; Angrist and Evans 1998; Hirvonen 2009; Lundborg et al. 2014). However, none of these studies observed labor supply more than 20 years after childbirth. To the best of our knowledge, ours is the first study to do so. A main contribution of this study is to estimate labor supply effects over the whole working life—that is, for 40 years after the birth of a second child.

Second, we contribute to the IV literature on children and labor market outcomes by studying how childbearing affects women’s relative position in the labor market, both in the short and in the long run. A large literature has explored the effect of having children on women’s hourly wages relying mainly on correlations in the cross section or on panel data estimation (Abendroth et al. 2014; Aisenbrey et al. 2009; Angelov et al. 2016; Budig and England 2001; Gangl and Ziee 2009; Korenman and Neumark 1992; Staff and Mortimer 2012; Wilde et al. 2010).^1^ The only study, to our knowledge, that used IV estimation to track the impact of children on hourly wages over time is Lundborg et al. (2014), who followed women up until 10 years after IVF treatment and found lasting and slightly increasing effects of entry into motherhood on women’s hourly wages (at 13 % after 10 years).

Naturally, hourly wages are observed only for those who are employed. Because employment is affected by childbearing, selection into and out of the labor force may bias estimated effects on hourly wages. We complement the literature on “motherhood wage penalties” by introducing other career measures that are not conditional on employment, and we track these career effects over the whole 40-year course, enabling us to estimate whether there is the same catching-up effect on career measures as there seems to be on labor supply.

Furthermore, this study contributes in two main ways to the literature specifically investigating how women are underrepresented at the top (Adda et al. 2017; Bertrand and Hallock 2001; Bertrand et al. 2010; Goldin 2014). We divide the sample by education to investigate how children affect careers among women with and without a college education. Several panel data studies have found greater motherhood wage penalties for women with higher education (Cools and Strøm 2016; Wilde et al. 2010). Bertrand et al. (2010) investigated the fast divergence in wages among males and females with master’s degrees in business administration (MBAs). In a dynamic life cycle model, Adda et al. (2017) showed that considerations about future fertility influence women’s occupational sorting: stronger skill atrophy in more abstract occupations makes women who want to have children relatively more likely to work in routine and manual occupations. A third contribution of our study is to bring IV evidence on the differential effect of children on women’s wage trajectories, depending on their education.

A recent literature has explored the role of firms and labor market sorting in explaining the gender wage gap (Goldin 2014; Kleven et al. 2017). Many of the career outcomes that we investigate in this study are related to aspects of the firm, and a fourth contribution of our study is therefore to bring IV evidence on how childbearing affects both in what type of firm women are employed as well as their relative position within this firm.

## Data and Empirical Method

### Sample Construction

All the data used in this study are register data from Statistics Norway. Demographic registers link children to their parents and provide information on parents’ marital status. Because the instrumental variable—same-sex children—is defined only for parents of at least two children, we start with the population of all men and women who had their second child in the period 1970–2001 and who were aged 18–45 at the birth of the second child. We include all parents of two children in our sample (not only married or cohabiting parents). Children are assigned to their mother and father in separate steps, and the analysis for both men and women is based on the birth of their own second child (as opposed to the second child of the specific couple). We have approximately 640,000 observations of second births for both men and women over the period 1970–2001. The number of second births is stable at approximately 20,000 per year. We use the same samples for the OLS and IV estimations throughout the study.

### IV Estimation

We aim to estimate the causal effect of individuals’ number of children (*C*) on their labor market outcomes (*Y*), as specified in Eq. (1):

1 $$ {Y}_i=\upalpha +\upbeta {C}_i+{\mathbf{X}}_i\mathbf{b}+{u}_i. $$

Even when we condition on a set of observable characteristics of the individual (**X**), the decision to have more children is likely to be endogenous to outcomes related to the labor market. We therefore use the event of parents having first and second children of the same sex as exogenous variation in their total number of children, exploiting the fact that parents with same-sex children have a higher probability of having a third child (Angrist and Evans 1998).

Estimating Eq. (1) by two-stage least squares (2SLS), the first stage is given by

2 $$ {C}_i=\upgamma +\updelta {Z}_i+{\mathbf{X}}_i\mathbf{d}+{\upupsilon}_i, $$

where *Z* is a dummy variable for individual *i*’s second child being of the same sex as the first child.

The first stage estimates show that 10 years after the birth of the second child, parents of two children of the same sex have, on average, approximately 0.07 more children than parents of two opposite-sex children. Most commonly, the increase in family size concerns only a third child, but a significant share also later have a fourth child and even higher-order births.^2^ In our main analysis, we divide the full sample into subgroups based on educational length. The first stage is stable across subsamples (see Table S2, Online Resource 1), and the *F* statistic usually lies around 1,000—well above conventional requirements for instrument relevance.

Because we are interested in how the effects on labor market outcomes evolve over time, the model is estimated separately for each year following the birth of the second child. The birth of the second child serves as our starting point (*t*_0_) because this is when the instrumental variable is realized, and it is a point in time that is defined for the whole sample. The birth year of a possible third child, on the other hand, is defined for only those who have additional children. The parameter of interest is β, which measures the effect of family size on the outcome in question a given number of years after *t*_0_. To increase the precision of the estimates, we also use 10-year averages of the outcome variables. We then estimate separate effects for 1–10, 11–20, 21–30, and 31–40 years after the birth of the second child.

The smaller first-stage coefficients during the first years after *t*_0_ reflect that the spacing between the second and the third child varies (from approximately 1 year to approximately 10 years). Because of a weak first stage during the first 3 years, we do not report the second-stage effects for these early years separately. The first 3 years are, however, included in the aggregate estimates for the first 10 years after the birth of the second child. There are fewer observations during the last 10-year period—31–40 years after *t*_0_—which substantially increases standard errors.

In all the estimations presented in this article, the set of control variables (**X**) includes dummy variables for the parent’s age, educational level, marital status, and country of origin; a linear control for income; dummy variables for the sex of the first and for the second child; a dummy variable for IVF treatment; and dummy variables for the birth year of the second child. All the control variables are observed in the year during which the individual has his/her second child, except income, which is the maximum of the individual’s yearly income during the two years before.

Summary statistics for the background characteristics are reported in Table 1, separately for women and men and for the value of the instrumental variable *Z*. Two-sided *t* tests indicate that there are no significant differences in the observable background characteristics between those who have two different-sex children and those who have two same-sex children (*p* values reported in separate columns). Thus, the sex mix of the two first children appears to result from a natural experiment, unrelated to observable characteristics that also influence labor market outcomes.

**Table 1:** Descriptive statistics

	Women					Men				
	*Z* = 0		*Z* = 1			*Z* = 0		*Z* = 1		
	Mean	SD	Mean	SD	Diff. *p*	Mean	SD	Mean	SD	Diff. *p*
Age	28.2	4.46	28.2	4.45	.73	30.7	4.74	30.7	4.75	.10
Years of Schooling	12.2	2.72	12.2	2.72	.89	12.6	2.93	12.6	2.90	.27
Married	0.15	0.36	0.15	0.36	.44	0.15	0.35	0.15	0.35	.55
Region of Origin										
Western Europe and North America	0.061	0.24	0.060	0.24	.20	0.057	0.23	0.057	0.23	.57
East and Central Europe	0.0054	0.073	0.0052	0.072	.18	0.0062	0.078	0.0058	0.076	.06
Africa	0.0048	0.069	0.0048	0.069	.77	0.0066	0.081	0.0063	0.079	.16
Asia	0.018	0.13	0.018	0.13	.95	0.017	0.13	0.017	0.13	.26
South America	0.0019	0.043	0.0019	0.044	.61	0.0020	0.045	0.0020	0.045	.82
Earnings	2.48	2.18	2.48	2.16	.77	5.80	3.32	5.83	10.90	.19
IVF Treatment	0.0022	0.047	0.0023	0.048	.38	0.0027	0.051	0.0029	0.054	.09
*N*	318,144		318,890			305,040		305,508		

*Note:* The sample is all men and women who had a second child in Norway between 1970 and 2001.

In addition to instrument relevance (as documented by the first-stage estimates) and random allocation (as documented by the *t* tests in Table 1), internal validity of the instrumental variable requires that children’s sex mix has no effect of its own on labor market outcomes, apart from the one working through an effect on family size. Importantly, this criterion regards direct effects of the children’s sex composition. The separate effect of having a boy compared with having a girl is controlled for by the two dummy variables for the sex of the first and the second child included in **X**. Bütikofer (2011) found no evidence of economies of scale for families with same-sex children in richer countries (a concern raised by Rosenzweig and Wolpin 2000:832). Huber (2015) did not find evidence of a violation of the exclusion restriction for the same-sex instrument in the data used by Angrist and Evans (1998). In addition, Angrist et al. (2010), as well as Cools and Hart (2015) in the Norwegian setting, found no evidence of direct effects of sex composition in samples for which sex composition did not affect family size.

A last standard requirement for instrument validity is *monotonicity*: that having two same-sex children never induces someone to actually refrain from further childbearing (Imbens and Angrist 1994). Finding the same-sex instrument to violate this assumption, Chaisemartin (2014) proposed a weaker requirement, under which IV “defiers” must be matched in number by a subgroup of “compliers” with identical probability distributions of the potential outcomes. This assumption is found to hold with high probability for the same-sex instrument.

The effect estimated applying the same-sex IV captures the causal effect of family size for individuals who decide whether to continue childbearing beyond their second child based solely on the sex mix of their two first children: the local average treatment effect (LATE).^3^ The effect cannot necessarily be generalized to other fertility margins or to individuals who are not influenced by children’s sex mix in their decision to have a third child. However, Angrist and Fernandez-Val (2010) found that differences in observable characteristics of parents who are influenced by the same-sex IV, relative to parents that are moved by the twin IV, explain most of the difference in the effect on labor supply as estimated by using the two different IVs.

### Outcome Variables

The long time span of the demographic data allows us to estimate the effect on labor market outcomes up until 40 years after the birth of the second child. Several outcome variables are reliably observed only in the period 1992–2010, and we have therefore restricted all observations of outcomes to this period of analysis.

For each outcome, we estimate the model presented earlier for yearly measures of the outcome variables and for 10-year averages: the 1–10, 11–20, 21–30, and 31–40 years after the second child is born. Descriptive statistics for the 10-year averages of all outcomes are given in Table S1 of Online Resource 1.

The restriction placed by the observation years (1992–2010) means that no individual will be in our data for more than 19 consecutive years, and those who have their second child after 1992 will be there for fewer. Hence, in the 10-year averages, not all individuals are observed in all 10 years. Because the 10-year averages that are based on relatively fewer yearly observations are relatively more noisy, we weigh the observations with the number of observation years underlying the measure when estimating the model for these 10-year outcomes.

The outcome variables measuring earnings and labor supply are based on both income registers and the worker-employee register. Importantly, they are not conditional on employment, and we observe them every year for all individuals. There is therefore no potential selectivity of the sample for these outcomes. Yearly earnings (reported in 1,000 USD) are taken directly from the tax registers. They are first consumer price index (CPI) converted into 2015 NOK, and then converted to USD at 0.11554 USD/NOK. Finally, they are bottom-coded at 0 and top-coded at 30 BA.^4^ Employment at the extensive margin (labor force participation) is captured by a dummy variable indicating yearly earnings above 1 BA.

Total employment is measured by contracted weekly hours taken from the worker-employee register. Hours are set to 0 if the individual is not employed. The effect on employment at the intensive margin is therefore the effect on total weekly hours, in percentage terms, minus the effect on employment. The effect on hourly wages could also be found by subtracting, in percentage terms, the effect on total weekly hours from the effect on yearly earnings. When doing these calculations, one should however keep in mind that the measure on weekly hours concerns contracted hours only: overtime is not included. Hence, the measure may cause an underestimation of the reduction in hours upon having children (if overtime is reduced), and thereby the estimated effect on labor supply at the intensive margin may be biased upward, and the resulting calculation of the effect on hourly wages may be biased downward.

We have two sets of variables that capture career outcomes beyond labor supply. The first is a set of dummy variables indicating whether the individual is employed by a firm in which the average level of earnings paid out is above the 25th, 50th, and 75th percentiles of the distribution of firms, ranked by the average earnings of their employees. The distribution of firms based on average earnings uses the earnings of all employees in all firms (i.e., the population of employees in Norwegian firms) and not just the individuals in our sample. Effects on these measures tell us whether individuals work for a different type of company—in terms of prestige/ambition/productivity, as captured by average earnings—after having more children. These measures are also unconditional on employment: The unemployed and those employed in a firm below the 25th percentile make up the reference category.

The second set of career outcomes contains variables that are measured within the employing firm and are, hence, conditional on employment. First, a dummy variable is used for whether the individual is the top earner of the company (a proxy for being the top manager or boss at the firm). Second, we have a continuous measure for the individual’s earnings rank within the company (from 0 to 1). Third, we use mean earnings of all the workers at the firm (again including workers not in the sample, but excluding the earnings of the index person), which is thus a conditional version of the unconditional firm rank measures.

## The Effect of Additional Children on Labor Supply

In this section, we present estimates of the effect on labor supply, as captured by the variables described in the previous section: a dummy variable for employment, total number of hours worked per week, and total labor earnings measured in 1,000 USD.

**Fig. 1:**
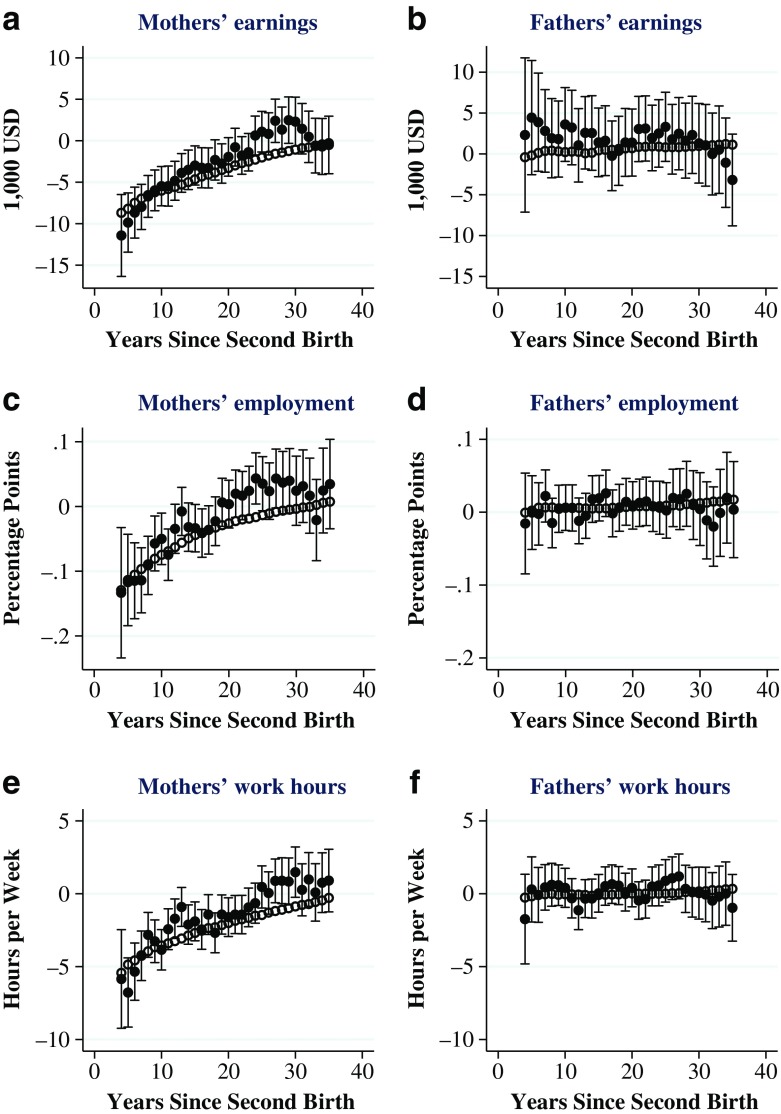
Effects of family size on parents’ labor supply. Each filled circle in the figure is the result from a 2SLS estimation of the impact of number of children on earnings and employment. The open circles are OLS estimates of the same relationship. The sample for both estimations is all men and women who had a second child in Norway between 1970 and 2001. Error bars show 95 % confidence intervals

Yearly estimates of the effects on labor supply for the whole sample are reported in Fig. 1. Each filled (black) circle in the graph gives the 2SLS estimate of the effect of number of children, estimated for each year following the birth of the second child (indicated on the horizontal axis). For comparison, empty (white) circles give the OLS estimates. The same sample is used for the OLS and 2SLS estimations (see the earlier section, Sample Construction, for a description). Because of a weak first stage during the first three years, we have not included the estimates for these years in the figure.

Table 2 displays the estimated effects on women’s labor supply outcomes, in 10-year aggregates. OLS estimates are presented in the four first columns, and IV estimates are in the four last columns. It is obvious from the OLS estimates that mothers with more than two children generally earn and work significantly less than mothers with exactly two children. The IV estimates suggest that this difference largely corresponds to a causal effect of children during the first 20 years after the birth of the second child. An additional child causes a 8.1 percentage point reduction in employment during the first 10 years (*p* < .01), and a 2.7 percentage point reduction during the next 10 years (*p* < .05).^5^

**Table 2:** The effect of number of children on women’s labor supply and earnings

	OLS				IV			
	1–10	11–20	21–30	31–40	1–10	11–20	21–30	31–40
All								
Employment	–0.11** (0.0012)	–0.043** (0.00063)	–0.013** (0.00084)	0.0056** (0.0013)	–0.081** (0.025)	–0.027* (0.013)	0.028^†^ (0.017)	0.019 (0.025)
Work hours	–4.88** (0.041)	–2.52** (0.022)	–1.34** (0.027)	–0.34** (0.039)	–5.00** (0.82)	–1.82** (0.44)	–0.25 (0.55)	0.56 (0.76)
Earnings	–7.55** (0.073)	–4.43** (0.044)	–2.02** (0.051)	–0.37** (0.067)	–7.93** (1.48)	–3.53** (0.88)	0.60 (1.02)	0.10 (1.33)
*N*	370,316	530,199	393,818	212,025	370,316	530,199	393,818	212,025
Work hours if employed	–3.18**	–1.84**	–1.37**	–0.90**	–3.07**	–1.44**	–0.98*	–0.10
	(0.045)	(0.018)	(0.021)	(0.039)	(0.91)	(0.36)	(0.39)	(0.70)
*N*	199,471	371,500	249,029	84,048	199,471	371,500	249,029	84,048
No College								
Employment	–0.13** (0.0016)	–0.052** (0.00076)	–0.017** (0.00096)	0.0019 (0.0014)	–0.090** (0.032)	–0.033* (0.015)	0.040* (0.019)	0.044 (0.027)
Work hours	–5.22** (0.049)	–2.75** (0.026)	–1.47** (0.031)	–0.50** (0.042)	–5.09** (0.98)	–1.85** (0.52)	0.039 (0.61)	1.02 (0.84)
Earnings	–7.28** (0.077)	–4.56** (0.046)	–2.18** (0.054)	–0.60** (0.069)	–5.79** (1.56)	–2.55** (0.91)	1.51 (1.07)	1.00 (1.37)
*N*	266,834	410,060	324,615	182,133	266,834	410,060	324,615	182,133
Hours if employed	–3.41**	–1.99**	–1.48**	–1.02**	–3.32**	–1.53**	–0.99*	–0.18
	(0.062)	(0.023)	(0.025)	(0.043)	(1.25)	(0.43)	(0.46)	(0.80)
*N*	123,708	269,352	194,539	71,166	123,708	269,352	194,539	71,166
College								
Employment	–0.051** (0.0019)	–0.011** (0.0010)	0.0051** (0.0015)	0.020** (0.0028)	–0.060 (0.038)	–0.015 (0.021)	–0.035 (0.032)	–0.15* (0.060)
Work hours	–4.21** (0.072)	–1.76** (0.041)	–0.71** (0.058)	0.31** (0.092)	–4.69** (1.46)	–1.97* (0.85)	–1.82 (1.19)	–2.32 (1.87)
Earnings	–8.53** (0.17)	–4.10** (0.11)	–1.48** (0.15)	0.14 (0.21)	–12.6** (3.38)	–7.06** (2.37)	–4.68 (3.07)	–5.83 (4.38)
*N*	103,482	120,139	69,203	29,892	103,482	120,139	69,203	29,892
Hours if employed	–3.10**	–1.58**	–1.01**	–0.31**	–3.10*	–1.24*	–0.91	0.30
	(0.065)	(0.030)	(0.039)	(0.088)	(1.30)	(0.61)	(0.74)	(1.43)
*N*	75,763	102,148	54,490	12,882	75,763	102,148	54,490	12,882

*Notes*: Each cell displays the result of a separate OLS or 2SLS estimation of the effect of number of children on women’s labor market outcomes. The outcome variable is indicated by the row header, and the time interval during which it is measured is indicated by the column header (the number in the header measures years since the birth of the second child). The sample in the top panel is all women in Norway who gave birth to a second child between 1970 and 2001. The next two panels show results from splitting this sample according to college education. Standard errors are shown in parentheses.

^†^ *p* < .10; *p < .05; **p < .01

The fall in labor force participation is reflected in significant drops in both work hours and earnings during these periods. Weekly work hours are, on average, reduced by 5 hours per week during the first 10 years and by 1.8 hours per week during the next 10. Compared with the mean working hours in the respective periods (20.2 and 23.8), this means a reduction of 24.7 % and 7.6 % in total, thus exceeding the drop in employment and indicating an even greater effect on labor supply along the intensive margin. At the bottom of each panel in Table 2, we show the estimated effect on work hours conditional on employment. The results show clearly that the effect on work hours is not entirely driven by movements in employment at the extensive margin. Conditional work hours are significantly reduced during the first 30 years (even after labor supply at the extensive margin is restored).

The reduction in earnings constitutes approximately 25 % of the mean during the first 10-year period and approximately 9 % during the next. Because these numbers are slightly larger than the percentage reduction in labor supply, they suggest that hourly wages also may be affected. We do not observe hourly wages directly, but the effect on hourly wages can be calculated from the effects on earnings and hours (using bootstrapping methods to obtain standard errors). In Fig. S1 in Online Resource 1, we show the results of this exercise. The estimates are imprecise but are consistently negative for women with college the first 20 years and mostly negative after. In the next section, we further investigate the effect of children on career outcomes, using alternative measures.

Figure 1 shows that there are no significant effects on men’s labor supply, as measured by any of the variables. This also holds when the outcomes are aggregated. These estimates for men are therefore not displayed (available upon request). In the long run, women’s earnings and labor supply are no longer negatively affected by the additional child. Although long-lasting, the drop in labor supply is eventually restored and related to when the children are still living with their parents.

The lower panels of Table 2 display separate estimates for subsamples according to whether the women in question have (at least) a college education. There are some notable differences according to educational status in the effect of childbearing on labor supply. Research in other settings has documented that women with relatively less education tend to exit the labor market to a larger extent in response to having an additional child (Angrist and Evans 1998; Hirvonen 2009; Maurin and Moschion 2009). In our setting, the effect on labor force participation is largely driven by the sample of non–college-educated women. Although the difference in point estimates is not statistically significant, it is worth bearing in mind that women in the group with relatively less education start out with a much lower level of employment. In the long run (21–40 years after the second child is born), the point estimates are actually significantly more positive for women without a college degree than for women with a college degree. Note, however, that we observe only one cohort—women who gave birth in the 1970s—for the last 10-year period. Therefore, for the longest-run outcomes, we cannot separate the long-term effect from a cohort effect. In addition, splitting the sample by education may increase the difference between earlier and later cohorts, given that there has been a significant increase in women’s education level over the period. Additional analyses in which we split the sample according to the decade in which the second child was born, show the same tendency of recovering labor supply for all cohorts as well as greater reductions in labor supply in the short run for women without college (see Table S3 of Online Resource 1).

In absolute size, the short- and medium-run effect on total hours is similar for women in both education groups; as a percentage of the mean level of working hours, however, the effect is larger for the lower-educated. The effect on college-educated women constitutes a reduction of 8.6 % relative to total hours, but this ratio is 27.6 % for women with no college degree. Having an extra child thus affects female part-time work in both education groups in addition to having an effect on employment.^6^

In their dynamic life cycle model, Adda et al. (2017:295) estimated that approximately one-fourth of the career costs of children stems from “wage responses, as a result of lost investments in skills and depreciation” (the rest is due to labor supply). Again, by comparing the estimated effect, in percentage terms, the estimates suggest a wage penalty of approximately 9 % for the college-educated women and a wage premium of 6 % for women without college. As discussed in the previous section, measured total hours count only contracted hours. Hence, if the amount of overtime is reduced by having an additional child, we underestimate the effect on hours and, as a consequence, the effect on hourly wages will be biased downward. In the next section, we investigate alternative outcomes related to career and job characteristics, which do not suffer from the same bias.

## The Effect of Additional Children on Career Outcomes

In Table 3, we estimate the effect of family size on the probability of being employed by a firm in which the average salary lies above a certain quartile in the distribution of all firms’ average salaries. The probabilities are unconditional on being employed (see the earlier section, Outcome Variables, for a description of the variables). Looking at the sample as a whole, there are negative effects on being employed by a firm in any part of the distribution in the short to medium run. The probability of working at a firm with average earnings above the 75th percentile falls by 4.6 percentage points in the first 10-year period and by 2.2 percentage points in the next, indicating substantial career effects of having an extra child. However, the effects differ substantially according to education category. Looking at the whole distribution, effects are situated in the lower part and wane in the long run for women without college, whereas they are both long-lasting and situated in the upper part of the distribution for college-educated women.^7^ For the latter group, the probability of working in a high-paying company (above the 75th percentile) is reduced by 7–8 percentage points throughout their career.

**Table 3:** The effect of number of children on women’s employing firm’s payment rank

	OLS				IV			
	1–10	11–20	21–30	31–40	1–10	11–20	21–30	31–40
All								
Employer above 25th percentile	–0.12**	–0.051**	–0.025**	–0.0016	–0.13**	–0.035*	0.00034	0.014
	(0.0014)	(0.00071)	(0.00088)	(0.0012)	(0.028)	(0.014)	(0.017)	(0.024)
Employer above median	–0.11**	–0.064**	–0.049**	–0.033**	–0.079**	–0.043**	0.0053	–0.00084
	(0.0015)	(0.00079)	(0.00090)	(0.0011)	(0.030)	(0.016)	(0.018)	(0.022)
Employer above 75th percentile	–0.069**	–0.046**	–0.043**	–0.030**	–0.044^†^	–0.021	–0.015	–0.013
	(0.0012)	(0.00064)	(0.00068)	(0.00073)	(0.025)	(0.013)	(0.014)	(0.014)
*N*	370,316	530,199	393,818	212,025	370,316	530,199	393,818	212,025
No College								
Employer above 25th percentile	–0.14**	–0.062**	–0.030**	–0.0070**	–0.15**	–0.039*	0.0079	0.041
	(0.0017)	(0.00085)	(0.00100)	(0.0014)	(0.034)	(0.017)	(0.020)	(0.027)
Employer above median	–0.11**	–0.070**	–0.054**	–0.038**	–0.075*	–0.030^†^	0.014	0.021
	(0.0017)	(0.00089)	(0.00099)	(0.0012)	(0.034)	(0.018)	(0.020)	(0.023)
Employer above 75th percentile	–0.062**	–0.045**	–0.044**	–0.031**	–0.029	–0.0071	–0.0022	–0.0028
	(0.0013)	(0.00069)	(0.00073)	(0.00079)	(0.027)	(0.014)	(0.014)	(0.016)
*N*	266,834	410,060	324,615	182,133	266,834	410,060	324,615	182,133
College								
Employer above 25th percentile	–0.078**	–0.018**	0.000025	0.020**	–0.097*	–0.032	–0.042	–0.15*
	(0.0022)	(0.0012)	(0.0017)	(0.0028)	(0.045)	(0.026)	(0.036)	(0.060)
Employer above median	–0.10**	–0.046**	–0.029**	–0.010**	–0.085	–0.092**	–0.045	–0.14*
	(0.0029)	(0.0016)	(0.0021)	(0.0029)	(0.058)	(0.034)	(0.044)	(0.061)
Employer above 75th percentile	–0.088**	–0.051**	–0.043**	–0.026**	–0.076	–0.073*	–0.086*	–0.079^†^
	(0.0028)	(0.0016)	(0.0018)	(0.0020)	(0.056)	(0.032)	(0.037)	(0.041)
*N*	103,482	120,139	69,203	29,892	103,482	120,139	69,203	29,892

*Notes:* Each cell displays the result of a separate OLS or 2SLS estimation of the effect of number of children on women’s probability of being employed by a firm in a specific segment of the firms’ average payment distribution. The outcome variable is indicated by the row header, and the time interval during which it is measured is indicated by the column header (the number in the header measures years since the birth of the second child). The sample in the top panel is all women in Norway who gave birth to a second child between 1970 and 2001. The next two panels show results from splitting this sample according to college education. Standard errors are shown in parentheses.

^†^ *p* < .10; *p < .05; **p < .01

The estimated effects on employment in Tables 2 and 3 are given in percentage points. Because employment rates are not uniform, neither across education groups nor across the distribution of firms by their average salaries, we need to compare these percentage point estimates with the corresponding average employment in order to know how relatively important the effects are. Table 4 shows the ratio between the percentage point estimates from Tables 2 and 3 and the average employment in that segment (displayed in Table S1, Online Resource 1) calculated by dividing the former by the latter. The comparison of the coefficients to average employment rates reveals that employment in higher-paying firms is disproportionately affected for the college-educated. Across education groups, the statistically significant effects are almost symmetrically situated around the diagonal. Effects among women with no college are concentrated in the lower part of the distribution and in the short run; for women with college, the largest effects are in the long run and in the higher parts of the distribution. Again, the difference in the size of the coefficients is striking.

**Table 4:** Effects on firm’s payment rank in percentage of mean employment

	IV			
	1–10	11–20	21–30	31–40
All				
Employed	–0.11**	–0.03*	0.04^†^	0.04
Employed by firm above 25th percentile	–0.21**	–0.05*	0.00	0.00
Employed by firm above median	–0.18**	–0.09*	0.01	–0.01
Employed by firm above 75th percentile	–0.26^†^	–0.12^†^	–0.08	–0.13
No College				
Employed	–0.12**	–0.03*	0.04*	0.04
Employed by firm above 25th percentile	–0.25**	–0.06*	0.01	0.09
Employed by firm above median	–0.21*	–0.07	0.04	0.08
Employed by firm above 75th percentile	–0.23	–0.05	–0.01	–0.02
College				
Employed	–0.07	–0.02	–0.04	–0.24*
Employed by firm above 25th percentile	–0.12*	–0.04	–0.05	–0.26*
Employed by firm above median	–0.13	–0.13**	–0.07	–0.33*
Employed by firm above 75th percentile	–0.27	–0.26*	–0.34*	–0.64*

*Note:* Each cell displays the ratio from taking the estimate from the 2SLS estimations displayed in Table 3 and dividing it by the average employment in that segment (displayed in Table S1 in Online Resource 1).

^†^ *p* < .10; *p < .05; **p < .01

These unconditional probabilities of being employed by a firm in a given segment also capture effects on labor force participation. In Table 5, we show estimates of the effect of family size on career outcomes among women who remain employed. There is no consistent effect on the conditional likelihood of being a top earner/boss, but there are negative effects on the within-firm rank.^8^ This effect is strongest—at a 13 percentage point reduction—for college-educated women in the short run (1–10 years), after which it disappears. For women with no college education, the initial effect is somewhat smaller (at 3–4 percentage points), but it lasts longer (1–20 years). In accordance with the unconditional estimates in Table 3, having an extra child means being employed by a firm with lower mean earnings, especially for college-educated women.

**Table 5:** The effect of number of children on women’s career measures (conditional on employment)

	OLS				IV			
	1–10	11–20	21–30	31–40	1–10	11–20	21–30	31–40
All								
Highest earnings	–0.012**	–0.0073**	–0.0030**	–0.00039	–0.0065	–0.0050	0.014	–0.0046
	(0.00062)	(0.00038)	(0.00044)	(0.00061)	(0.012)	(0.0075)	(0.0085)	(0.011)
Earnings rank	–0.059**	–0.042**	–0.012**	0.0077**	–0.070**	–0.023^†^	0.021	–0.012
	(0.0011)	(0.00071)	(0.00083)	(0.0012)	(0.022)	(0.014)	(0.016)	(0.020)
Mean earnings	–3.99**	–2.84**	–2.69**	–2.90**	–2.25	–1.50*	–1.42^†^	–1.30
	(0.079)	(0.038)	(0.040)	(0.062)	(1.56)	(0.75)	(0.77)	(1.06)
*N*	317,505	453,621	318,687	129,803	317,505	453,621	318,687	129,803
No College								
Highest earnings	–0.012**	–0.0071**	–0.0029**	–0.00012	–0.0051	–0.013	0.0066	0.00035
	(0.00072)	(0.00041)	(0.00046)	(0.00065)	(0.014)	(0.0080)	(0.0089)	(0.011)
Earnings rank	–0.047**	–0.036**	–0.0096**	0.0079**	–0.043^†^	–0.032*	0.014	–0.016
	(0.0012)	(0.00076)	(0.00088)	(0.0012)	(0.023)	(0.015)	(0.017)	(0.022)
Mean earnings	–3.97**	–2.98**	–2.88**	–3.07**	–2.33	–0.48	–1.08	–0.85
	(0.094)	(0.043)	(0.046)	(0.070)	(1.82)	(0.83)	(0.88)	(1.19)
*N*	220,261	342,233	256,438	108,110	220,261	342,233	256,438	108,110
College								
Highest earnings	–0.012**	–0.0080**	–0.0045**	–0.0024	–0.010	0.020	0.044^†^	–0.031
	(0.0012)	(0.00089)	(0.0012)	(0.0016)	(0.024)	(0.018)	(0.024)	(0.031)
Earnings rank	–0.081**	–0.059**	–0.023**	0.0017	–0.13*	0.0042	0.048	0.0063
	(0.0025)	(0.0017)	(0.0022)	(0.0030)	(0.049)	(0.035)	(0.044)	(0.057)
Mean earnings	–4.20**	–2.59**	–2.15**	–2.16**	–2.15	–4.53**	–3.16^†^	–3.72^†^
	(0.15)	(0.081)	(0.083)	(0.12)	(2.91)	(1.67)	(1.62)	(2.25)
*N*	97,244	111,388	62,249	21,693	97,244	111,388	62,249	21,693

*Notes:* Each cell displays the result of a separate OLS or 2SLS estimation of the effect of number of children on women’s career outcomes. The outcome variable is indicated by the row header, and the time interval during which it is measured is indicated by the column header (the number in the header measures years since the birth of the second child). The sample in the top panel is all women in Norway who gave birth to a second child between 1970 and 2001. The next two panels show results from splitting this sample according to college education. Standard errors are shown in parentheses.

^†^ *p* < .10; *p < .05; **p < .01

If women whose career is more negatively affected are more likely to exit the labor force, the estimates in Table 5 are biased upward. This is one possible explanation for the relative lack of effects on earnings rank, especially among college-educated women, compared with the unconditional measures in Table 3. Another explanation is that college-educated women more often move to a different job. A possible scenario is that for women with a college degree, having an additional child causes a setback in the company where they work. They then move to a lower-paying, possibly less-prestigious, company—where they retain their rank within the firm and where they might even have a slightly higher chance of being the top earner/boss. There does not seem to be a similar pattern for women without college. They lose out in relative rank within the firm during the first 20 years after the second child is born, but there is no evidence that they change employer type (in terms of mean earnings by the other employees) in the longer run.

## Conclusion

Parenthood is generally considered to have severe and long-lasting consequences for women’s careers, even after they return to full time work. Nevertheless, women’s career paths may be less steep than men’s even before having children (Manning and Swaffield 2008), and women who intend to have (relatively more numerous) children may, on average, give less priority to succeeding in the labor market than women with no (or fewer) children. Therefore, the exact effect of childbearing and child rearing on women’s careers is not known merely from comparing women with children to women without children, or from comparing women with different number of children to each other.

In this study, we address these concerns about causality by offering evidence on the career costs of children using IV estimation. We employ a well-known instrumental variable for number of children—the sex composition instrument—which induces families to have more children based only on the sex mix of the first two children. Because children’s sex mix is randomly allocated in our sample, we can use this variation to estimate the difference in career paths for women with two children and women who have more than two children, without the concerns about selection and confounding factors that apply estimates obtained by OLS or fixed-effects estimation.

In line with similar studies from other settings (Angrist and Evans 1998; Cruces and Galiani 2007), our findings show significant negative effects of having additional children on labor supply, at both the extensive and the intensive margin in the short and medium run. Adding to this literature, we present new evidence that in the longer run, from 20 years after the second child is born and onward, labor supply is indeed restored, on average. Among women without college, having the additional child even seems to increase labor force participation.

Further adding to this literature, which mainly focuses on effects on labor supply, we find clear evidence that having additional children impedes women’s careers, and that these effects are concentrated among college-educated women. The effects on career outcomes are more persistent. Regardless of education level, women’s relative earnings within the firm fall in the short run. Although this is restored in line with labor supply in the short run for women without a college education, college-educated women have a smaller probability of being employed in a high-paying firm throughout the rest of their career. In addition, their relative earnings within the company are negatively affected in the short run.

Our findings are in line with Lundborg et al. (2014), who found a 13 % reduction in women’s hourly wages due to having a first child after 10 years, and with a large panel data literature on wage penalties to motherhood (e.g., Budig and England 2001; Harkness and Waldfogel 2003). However, we trace out effects on these career measures for much longer than any existing study that we are aware of, following women over their entire career. In addition, many of our career measures are unconditional on employment and therefore not suffering from bias due to selective labor market exit and entry.

We find no long-term effect on the probability of being the top earner or on the individual’s earnings rank within the firm. The results may therefore suggest that family size mainly affects the type of firm in which a woman works, with women who have more children working in less-prestigious places of work but having the same within-firm responsibilities as women with fewer children. This is in line with the recent literature on the roles of firm attributes and labor market sorting on the gender wage gap (Goldin 2014; Kleven et al. 2017). To the best of our knowledge, there is no IV evidence of how having children affects type of employment for women, and there is little evidence of any kind regarding effects in the very long run.

For women without a college education, much weaker evidence suggests that family size hurts career outcomes. Our findings thus seem to corroborate the finding that having children and experiencing periods of reduced labor supply is more harmful to careers in relatively more high-skilled jobs (Adda et al. 2017; Bertrand et al. 2010; Cools and Strøm 2016; Wilde et al. 2010).

This article adds to the knowledge of the extent to which motherhood comes in conflict with labor market ambitions. We see that even beyond the second parity, there are additional penalties to women’s careers. The declining number of families who have more than two children—rather than a drop in first and second births—is argued to have caused fertility declines in Western countries to below-replacement level (Morgan 2003). Given that women in these countries, on average, state that their preferred number of children is two (Bongaarts 2002), the decision to move beyond a second birth is highly likely to be influenced by the perceived cost of additional children. In countries where levels of female labor force participation are high and the returns to human capital continue to rise, career costs may be among the most important considerations in this decision.

## Electronic supplementary material

ESM 1 (PDF 175 KB)

## Footnotes

1 With panel data, the causal interpretation of the estimations rests on the assumption that all unobserved heterogeneity is time-invariant, yet several studies using fertility shocks to instrument the timing of children have shown notable positive career effects of postponed birth (Hotz et al. 1997, 2005; Miller 2011). This suggests that the timing decision is likely to be influenced by career concerns.

2 The average number of children for parents whose first two children are of opposite sex is 2.48, compared with 2.55 for parents whose first two children are of the same sex. Further, 39 % of parents of opposite-sex children have a third child, compared with 44 % of parents of same-sex children. If we were rather to use the probability of a having third child as the endogenous regressor, the (long-term) first stage would lie slightly below 0.06.

3 Under Chaisemartin’s (2014) weaker assumption, the estimates are to be interpreted as the LATE not for the complier subpopulation as a whole but instead for the “surplus” group of compliers.

4 A base amount, BA, is used in the Norwegian social insurance system to calculate various benefits, including sickness absence and pensions. It is adjusted yearly according to the general wage increase for wage earners. In 2013, 6 BA = 511,470 NOK ≈ 83,743 USD.

5 As a robustness check, we also estimated the model using logit and IV logit for the employment outcome. The results (available upon request) were very similar.

6 According to the OECD, Norwegian women have among the highest rates of part-time work among member countries (OECD 2013).

7 Again, in the very long run (31–40 years after the birth of the second child), effects are estimated only for women who had their second child in the 1970s.

8 Because the probability of being the top earner is mechanically higher in smaller firms, we also estimated the effect of being in the top quartile of the intrafirm pay distribution. The results (available upon request) were not qualitatively different from the effect on being a top earner.
