# Supplementary material for: Children and Careers: How Family Size Affects Parents’ Labor Market Outcomes in the Long Run
Source: Demography. 2017 Sep 6;54(5):1773–93. doi: 10.1007/s13524-017-0612-0 (PMC5624987; doi:10.1007/s13524-017-0612-0)
Supplement: Supplementary file 1 — (PDF 175 KB) [file 13524_2017_612_MOESM1_ESM.pdf]

Online Resource 1

Children and Careers: How Family Size Affects Parents' Labor Market Outcomes in the Long Run

Sara Cools, Simen Markussen, Marte Strøm

Table S1: Descriptive statistics for all outcome variables.

	Women				Men			
	1-10	*11-20*	*21-30*	*31-40*	*1-10*	*11-20*	*21-30*	*31-40*
All								
Employment	0.75	0.82	0.76	0.53	0.92	0.89	0.79	0.51
Work hours	20.2	23.6	21.8	14.5	30.4	29.9	26.1	15.5
Earnings	3.16	3.85	3.55	2.33	6.66	6.88	5.94	3.45
Employer above 25p.	0.66	0.73	0.67	0.46	0.79	0.75	0.66	0.39
Employer above median	0.43	0.47	0.42	0.26	0.70	0.68	0.59	0.34
Employer above 75p.	0.18	0.19	0.16	0.09	0.46	0.44	0.37	0.20
N	370316	530199	393818	212025	351477	507880	381067	206220
Hours if empl.	27.2	29.1	29.3	28.2	33.3	33.9	33.5	31.6
Highest earnings	0.038	0.054	0.054	0.042	0.10	0.12	0.12	0.090
Earnings rank	0.15	0.25	0.25	0.19	0.40	0.46	0.42	0.30
Mean earnings	409	427	423	419	54.6	56.2	55.4	53.0
N	317505	453621	318687	129803	316630	428516	297733	113700
No college								
Employment	0.70	0.79	0.73	0.52	0.91	0.87	0.77	0.49
Work hours	18.3	22.2	20.6	14.0	29.8	29.1	25.1	14.7
Earnings	2.59	3.36	3.17	2.15	5.86	5.97	5.12	2.94
Employer above 25p.	0.60	0.69	0.64	0.44	0.76	0.72	0.62	0.37
Employer above median	0.35	0.41	0.37	0.24	0.66	0.63	0.55	0.31
Employer above 75p.	0.14	0.16	0.14	0.09	0.41	0.40	0.33	0.17
N	266834	410060	324615	182133	257868	382434	294992	163622
Hours if empl.	26.2	28.3	28.7	28.0	33.2	33.7	33.3	31.6
Highest earnings	0.036	0.049	0.049	0.040	0.096	0.11	0.10	0.079
Earnings rank	0.11	0.20	0.21	0.17	0.35	0.40	0.36	0.25
Mean earnings	381	406	410	410	512	532	530	518
N	220261	342233	256438	108110	228210	314537	222643	84925
College								
Employment	0.89	0.92	0.88	0.61	0.95	0.94	0.88	0.62
Work hours	25.2	28.4	27.2	17.0	31.8	32.1	29.5	18.8
Earnings	4.62	5.55	5.31	3.39	8.89	9.65	8.74	5.41
Employer above 25p.	0.81	0.86	0.82	0.54	0.87	0.85	0.78	0.50
Employer above median	0.64	0.69	0.67	0.42	0.81	0.80	0.73	0.46
Employer above 75p.	0.28	0.28	0.22	0.12	0.61	0.58	0.50	0.28
N	103482	120139	69203	29892	93609	125446	86075	42598
Hours if empl.	28.8	31.2	31.5	29.5	33.5	34.2	33.8	31.7
Highest earnings	0.043	0.068	0.078	0.055	0.11	0.15	0.16	0.12
Earnings rank	0.23	0.40	0.44	0.29	0.52	0.63	0.61	0.42
Mean earnings	473	490	477	464	634	643	623	597
N	97244	111388	62249	21693	88420	113979	75090	28775

Table S2: The effect of same sex on number of children. Effects on subgroups.

	Women				Men			
	1-10	*11-20*	*21-30*	*31-40*	*1-10*	*11-20*	*21-30*	*31-40*
Number of children								
All	.038***	.066***	.068***	.068***	0.038***	0.066***	0.068***	0.068***
	(0.0012)	(0.0018)	(0.0022)	(0.0029)	(0.0012)	(0.0018)	(0.0022)	(0.0029)
N	370316	530199	393818	212025	370316	530199	393818	212025
Lower level education	0.037***	0.066***	0.068***	0.068***	0.037***	0.066***	0.068***	0.068***
	(0.0014)	(0.0020)	(0.0024)	(0.0032)	(0.0014)	(0.0020)	(0.0024)	(0.0032)
N	266834	410060	324615	182133	266834	410060	324615	182133
Higher level education	0.038***	0.064***	0.066***	0.068***	0.038***	0.064***	0.066***	0.068***
	(0.0023)	(0.0038)	(0.0052)	(0.0080)	(0.0023)	(0.0038)	(0.0052)	(0.0080)
N	103482	120139	69203	29892	103482	120139	69203	29892

Figure S1: Effects on earnings, hours and hourly wages, for women without college (left) and women with college (right)

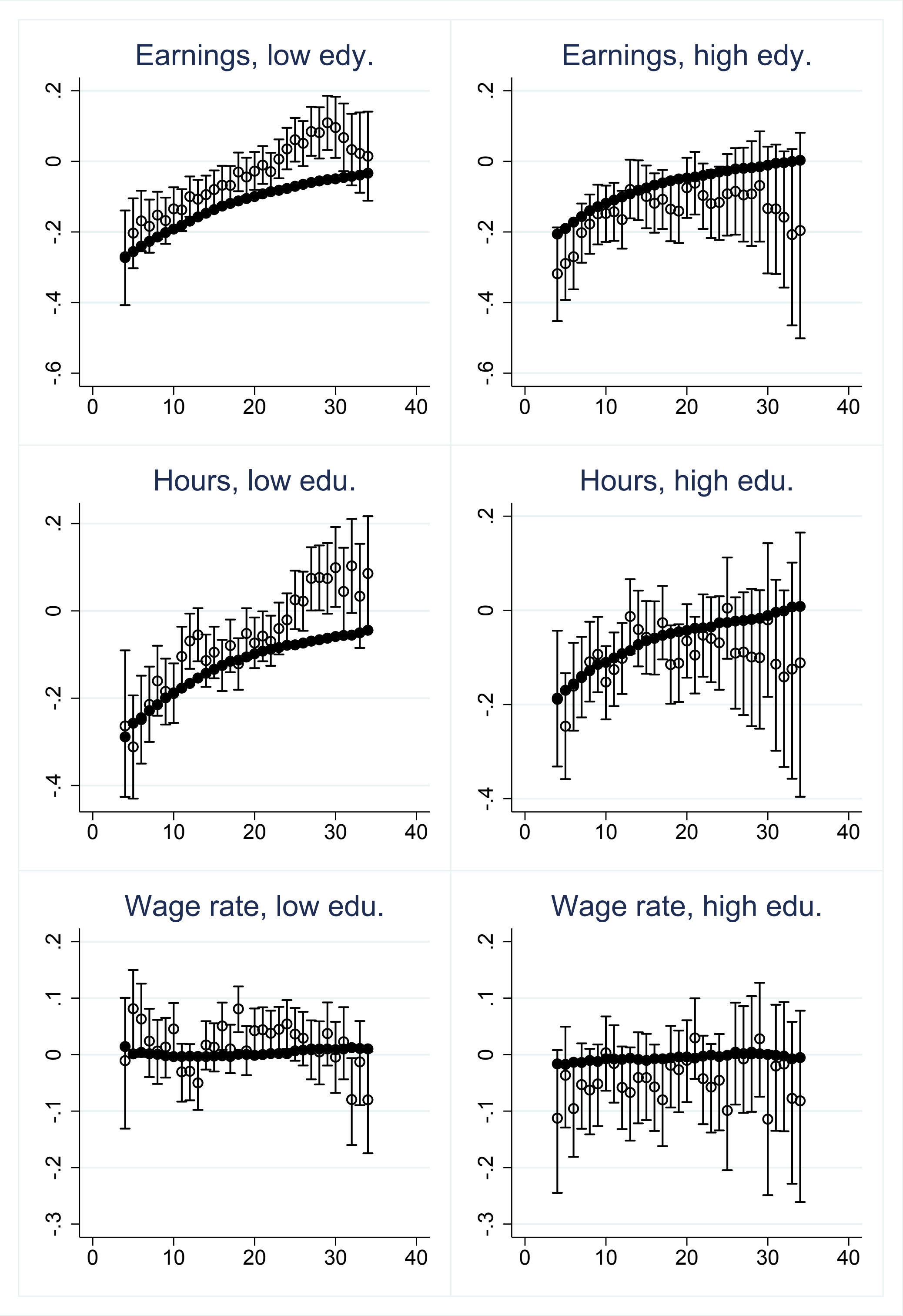

Note: Each open circle in the top and middle panel is the result from a 2SLS estimation of the impact of number of children on the outcome indicated in the header. The filled circles are the corresponding OLS estimates. Error bars give the 90% confidence interval for the estimate. The wage effects in the lower panel are calculated as percentages by log decomposing the change in earnings into a change in hours and a change in the wage rate ($log(Y'/Y) = log(W'/W) + log(H'/H)$, where Y is earnings, W is wage rate and H is hours, and for each variable, $X' = E[X] + \beta$). Confidence intervals are obtained by bootstrapping (4000 repetitions).

Table S3: The effect of number of children on women's labour supply and earnings, by birth cohort of the child

	All				No college				College			
	1-10	*11-20*	*21-30*	*31-40*	*1-10*	*11-20*	*21-30*	*31-40*	*1-10*	*11-20*	*21-30*	*31-40*
1970s												
Employment		-0.011	0.034	0.019		-0.0015	0.048*	0.044		-0.051	-0.049	-0.15**
		(0.027)	(0.023)	(0.025)		(0.031)	(0.026)	(0.027)		(0.043)	(0.046)	(0.060)
Work hours		-2.47***	-0.091	0.56		-2.43**	0.14	1.02		-2.66	-1.51	-2.32
		(0.92)	(0.73)	(0.76)		(1.04)	(0.81)	(0.84)		(1.79)	(1.67)	(1.87)
Earnings		-2.79*	0.16	0.10		-1.89	0.90	1.00		-7.55*	-4.57	-5.83
		(1.60)	(1.34)	(1.33)		(1.73)	(1.41)	(1.37)		(4.24)	(4.16)	(4.38)
N		144676	212025	212025		122901	182133	182133		21775	29892	29892
1980s												
Employment	-0.12***	-0.026	0.017		-0.14**	-0.033	0.024		-0.11*	-0.0079	-0.016	
	(0.046)	(0.020)	(0.024)		(0.056)	(0.023)	(0.027)		(0.068)	(0.034)	(0.047)	
Work hours	-5.68***	-1.82***	-0.50		-5.52***	-1.85**	-0.16		-7.17***	-1.97	-2.16	
	(1.44)	(0.67)	(0.83)		(1.71)	(0.76)	(0.94)		(2.63)	(1.37)	(1.76)	
Earnings	-8.69***	-2.74**	1.35		-7.24***	-2.14	2.58		-13.0**	-5.22	-4.93	
	(2.36)	(1.33)	(1.60)		(2.58)	(1.39)	(1.65)		(5.49)	(3.68)	(4.79)	
N	127100	181793	181793		98252	142482	142482		28848	39311	39311	
1990s												
Employment	-0.041	-0.033			-0.041	-0.045			-0.031	-0.0063		
	(0.033)	(0.023)			(0.041)	(0.028)			(0.050)	(0.033)		
Work hours	-4.21***	-1.52*			-4.15***	-1.50			-3.85**	-1.54		
	(1.07)	(0.80)			(1.27)	(0.98)			(1.90)	(1.33)		
Earnings	-7.13***	-5.05***			-5.16**	-3.49*			-11.9***	-8.87**		
	(1.97)	(1.73)			(2.06)	(1.79)			(4.47)	(4.05)		
N	203730	203730			144677	144677			59053	59053		
2000s												
Employment	-0.22***				-0.32***				-0.11			
	(0.082)				(0.12)				(0.10)			
Work hours	-7.92***				-10.4***				-5.25			
	(2.84)				(3.83)				(4.13)			
Earnings	-11.3**				-7.30				-16.1			
	(5.45)				(5.96)				(10.1)			
N	39486				23905				15581			

Note: Each cell displays the result of a separate OLS or 2SLS estimation of the effect of number of children on women's labour market outcomes. The outcome variable is indicated by the row header and the time interval during which it is measured is indicated by the column header (the number in the header measures years since the birth of the second child). The sample is all Norwegian women who gave birth to a second child between 1970 and 2001.
